# Diagnostic and Prognostic Value of Chest Radiographs for COVID-19 at Presentation

**DOI:** 10.5811/westjem.2020.7.48842

**Published:** 2020-08-17

**Authors:** Ariel Kerpel, Sara Apter, Noam Nissan, Esther Houri-Levi, Maximiliano Klug, Sharon Amit, Eli Konen, Edith M. Marom

**Affiliations:** * Department of Diagnostic Imaging, Sheba Medical Center, Tel Hashomer, affiliated with Sackler Faculty of Medicine, Tel Aviv University, Israel; § Department of Medicine ‘B’, Sheba Medical Center, Tel Hashomer, affiliated with Sackler Faculty of Medicine, Tel Aviv University, Israel; §§ Clinical Microbiology, Sheba Medical Center, Tel Hashomer, affiliated with Sackler Faculty of Medicine, Tel Aviv University, Israel

## Abstract

**Introduction:**

Pulmonary opacities in COVID-19 increase throughout the illness and peak after ten days. The radiological literature mainly focuses on CT findings. The purpose of this study was to assess the diagnostic and prognostic value of chest radiographs (CXR) for coronavirus disease 2019 (COVID-19) at presentation.

**Methods:**

We retrospectively identified consecutive reverse transcription polymerase reaction-confirmed COVID-19 patients (n = 104, 75% men) and patients (n = 75, 51% men) with repeated negative severe acute respiratory syndrome coronavirus 2 (SARS-CoV-2) tests. Two radiologists blindly and independently reviewed the CXRs, documented findings, assigned radiographic assessment of lung edema (RALE) scores, and predicted the patients’ COVID-19 status. We calculated interobserver reliability. The score use for diagnosis and prognosis of COVID-19 was evaluated with the area under the receiver operating characteristic curve.

**Results:**

The overall RALE score failed to identify COVID-19 patients at presentation. However, the score was inversely correlated with a COVID-19 diagnosis within ≤2 days, and a positive correlation was found six days after symptom onset.Interobserver agreement with regard to separating normal from abnormal CXRs was moderate (k = 0.408) with low specificity (25% and 27%). Definite pleural effusion had almost perfect agreement (k = 0.833) and substantially reduced the odds of a COVID-19 diagnosis. Disease distribution and experts’ opinion on COVID-19 status had only fair interobserver agreement. The RALE score interobserver reliability was moderate to good (intraclass correlation coefficient = 0.745). A high RALE score predicted a poor outcome (intensive care unit hospitalization, intubation, or death) in COVID-19 patients; a score of ≥5 substantially increased the odds of having a poor outcome.

**Conclusion:**

Chest radiography was found not to be a valid diagnostic tool for COVID-19, as normal or near-normal CXRs are more likely early in the disease course. Pleural effusions at presentation suggest a diagnosis other than COVID-19. More extensive lung opacities at presentation are associated with poor outcome in COVID-19 patients. Thus, patients with more than minimal opacities should be monitored closely for clinical deterioration. This clinical application of CXR is its greatest strength in COVID-19 as it impacts patient care.

## INTRODUCTION

Coronavirus disease 2019 (COVID-19) is spreading globally.^1^ The World Health Organization (WHO) declared COVID-19 a pandemic on March 11, 2020.^2^ The most common presenting clinical symptoms are fever, cough, dyspnea, myalgia, and fatigue.^3^–^5^ Older age and medical comorbidities are linked to more severe disease.^4^,^6^–^8^ Men are over-represented among COVID-19 patients.^3^,^4^,^6^,^7^

Although the radiological literature mainly focuses on computed tomography (CT) findings,^9^,^10^ many patients are imaged solely with chest radiography^10^,^11^ primarily as an adjunct to reverse transcription polymerase chain reaction (RT-PCR) but in some scenarios as a triage tool,^12^,^13^ especially in resource-constrained environments where the supply of laboratory PCR kits cannot meet the demand. Although there are nonspecific respiratory symptoms commonly observed in COVID-19 patients at presentation, some patients with COVID-19 do not present with these classic clinical manifestations, which further complicates triage and diagnosis.^4^

The chest radiograph (CXR) was reported as having a sensitivity of 69% for COVID-19 in one study of 64 patients.^9^ In that study, the common findings were bilateral peripheral opacities with a predilection to the lower lung zones. Opacities increased throughout the illness, with a peak in severity at 10–12 days after symptom onset; this was shown by documenting lung opacities using a simplified radiographic assessment of lung edema (RALE) score.^9^,^14^ When the Fleischner Society consensus statement was created, which specified that chest radiography has little value early in the course of the disease, there were limited data available on the accuracy of chest radiography for the diagnosis of COVID-19.^13^ Data on the strengths and weaknesses of chest radiography for the diagnosis of COVID-19 are important, as CXRs are the most commonly used triage imaging tool in any patient presenting with respiratory symptoms.^12^ This is especially important because experts suggest that the second wave of coronavirus is likely to be even more devastating.^15^

Our aim was to assess the diagnostic accuracy and reliability of CXRs in patients suspected of having COVID-19 at presentation to the emergency department (ED) and to assess the prognostic value of the RALE score in patients with COVID-19.

## MATERIALS AND METHODS

### Patients and Data Source

This retrospective study was approved by our institutional review board, and informed consent was waived. We identified our study population by extracting severe acute respiratory syndrome coronavirus 2 (SARS-CoV-2) RT-PCR test results (positive or negative) of nasopharyngeal swabs from all consecutive patients older than 18 years analyzed at our hospital’s laboratory from the ED from March 6–31, 2020, who had a CXR at presentation (within 24 hours of the first RT-PCR). We extracted data by a database search (query) using the MDClone platform (MDClone Ltd, Be’er Sheva, Israel), a big data system for healthcare. We were granted access to the raw data in order to validate the quality and reliability of the information in the database source underlying the study.

**Population Health Research Capsule**

What do we already know about this issue?

Pulmonary opacities in coronavirus disease 2019 (COVID-19) peak after 10 days. The radiological literature focuses on computed tomography findings.

What was the research question?

What is the diagnostic and prognostic value of chest radiographs (CXR) for COVID-19 at presentation?

What was the major finding of the study?

While CXR is not a valid diagnostic tool for COVID-19, the presence of extensive opacities is associated with poor outcome.

How does this improve population health?

CXR’s greatest strength in COVID-19 is prognosis prediction. Patients with more than minimal opacities should be monitored closely for clinical deterioration.

Of the RT-PCR test kits used, 90% (161/179) were Allplex 2019-nCoV assay kits (Seegene Inc. Seoul, Korea), and 10% (18/179) were kits produced in our hospital laboratory.

**Figure 1:**
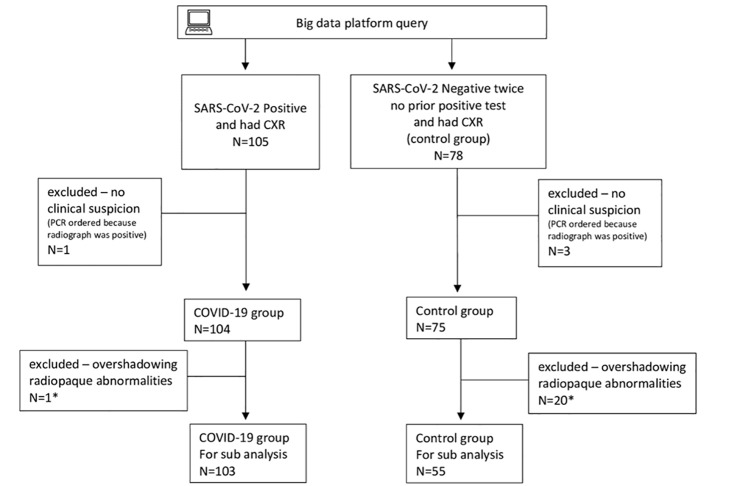
Study selection process flowchart. *Overshadowing radiopaque abnormalities excluded from the subgroup analyses were pleural effusion (n = 1 COVID-19, n = 17 control), lung cancer (n = 1, control), lung metastasis (n = 1, control), and pleural plaques (n = 1, control).

The patients were then divided into two groups: those who had COVID-19 and those who did not. The former group comprised patients who had a positive RT-PCR test. The latter, control group comprised patients who had a negative RT-PCR result on at least two separate occasions, more than 24 hours apart (without a previous positive test result). This methodology is similar to that of previously published studies,^16^ as we tried to avoid the imperfect gold standard bias. We excluded patients who underwent SARS-CoV-2 testing due to an abnormal CXR and not due to clinical suspicion (n = 1 positive, n = 3 negative) based on the patients’ electronic health records (EHR) (Figure 1) to avoid partial verification bias (referral bias).^17^

The patients’ EHRs were reviewed to obtain demographics and clinical data. The primary outcomes were intensive care unit (ICU) hospitalization, intubation, and mortality. COVID-19 severity was classified as severe or non-severe based on respiratory distress (≥30 breaths per minute) or oxygen saturation ≤93% on room air.^18^ Although lung opacities are included in some published severity criteria, we did not use CXR findings to define severity to avoid incorporation bias.^17^ The data cutoff date was April 21, 2020.

We extracted the overall number of ED visits at our hospital during the study period using the MDClone platform database search. Overall COVID-19 new cases in Israel for the study period (26 days), and for an equal time span before and after the study period, were extracted from Israel’s Ministry of Health website.^19^

### Imaging Protocols

CXRs were acquired as computed radiographs (n = 127) or digital radiographs (n = 52) from multiple vendors. The projections were posterior-anterior (PA) (n = 108), and anterior-posterior (AP) (n=71).

### Image Analysis

**Figure 2:**
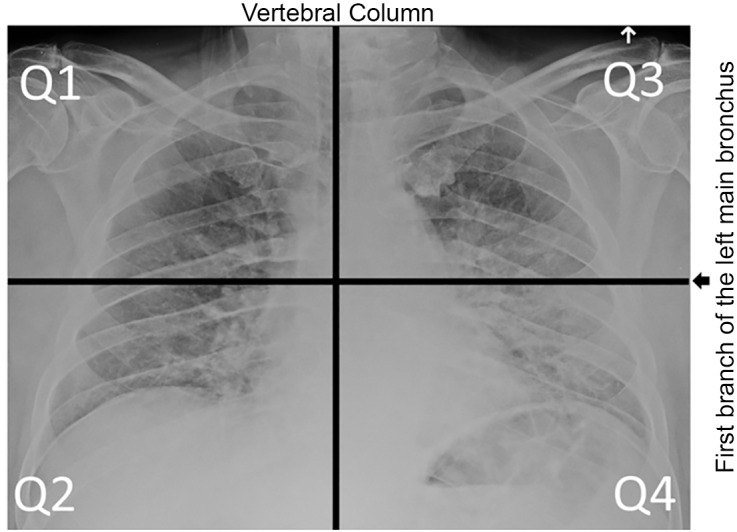
An example of radiograph assessment of lung edema (RALE) scoring in a 71-year-old man with COVID-19 who presented 5 days after symptom onset, with fever, cough and fatigue. RALE scoring: reader 1: 11, reader 2: 12. Adapted from Warren et al, 2018.^14^

Two radiologists (EMM, a thoracic radiologist with 28 years of experience, and SA, an oncology imaging radiologist with 40 years of experience) independently reviewed all CXRs using a communication system search (PACS), Carestream, PACS Vue v12.1.5 (Carestream Health, Inc, Rochester, NY), while blinded to the RT-PCR results and clinical data. The CXRs of COVID-19 patients and the control patients were in random order. Both readers recorded pulmonary opacity characteristics, including their distribution (peripheral, perihilar or diffuse), zonal predominance (upper, lower, or equal), and laterality (bilateral or unilateral). Pleural effusion presence was recorded. Disagreements between reader 1 (R1) and reader 2 (R2) regarding the categorization of a pleural effusion as definite or questionable were resolved by an independent and blinded third reader (EK, a cardiothoracic radiologist with 21 years of experience). R1 and R2 calculated the RALE scores^14^ (Figure 2). The RALE score, which is used to quantitate lung opacities,^14^ is calculated by dividing each radiograph into quadrants and multiplying the extent (0 = no involvement, 1 = <25%, 2 = 25–50%, 3 = 50–75%, 4 = >75%) by the density (1 = hazy, 2 = moderate, 3 = dense) for each quadrant and then summing them (maximum score = 48).^14^ For the purposes of our study, the following density definitions were used: hazy, ranging from barely noticeable opacities to mild or veiling opacities, through which the lung vessels can be clearly seen; moderate, in which opacities are identified, but the blood vessels are still visible; and dense, in which consolidation is apparent, and the blood vessels are not visible. For RALE scoring, we excluded CXRs with one of the following overshadowing radiopaque abnormalities: pleural effusion; pleural plaques; and pulmonary nodules or masses, whether due to lung cancer or metastatic disease.

Finally, the readers gave their expert opinion regarding patient COVID-19 status based on imaging alone. All previous imaging tests were available to the readers for comparison, and any changes were recorded.

### Statistical Analysis

To evaluate the sensitivity and specificity of categorical variables to discriminate between patients with and without COVID-19, assuming sensitivity and specificity of 80% and a 95% confidence interval (CI) of 0.2, 140 patients were needed. To evaluate the use of RALE for determining COVID-19 diagnosis using the area under the receiver operating characteristic curve (AUC), assuming an area of 0.8 with a 95% CI width of 0.2 and an equal number of participants with and without COVID-19, 78 participants were needed. We assumed that the mean RALE score for patients without poor prognosis was 2, with a mean score of 10 for patients with poor outcomes. We assumed that the standard deviation of the RALE score was 8 (range 0–48, divided by six). Using a significance level of 5% and power of 80%, and assuming a proportion of patients having poor outcomes to be 20%, a total of 53 patients were needed.

We evaluated continuous variables for normal distributions using histograms. Variables that were close to being normally distributed are reported as the means and standard deviations (SD), while skewed variables are reported as the medians and interquartile ranges. Categorical variables are reported as frequencies and percentages. We used independent samples t-tests and Mann–Whitney tests to compare normally distributed variables and skewed variables between groups, respectively. Chi-square tests and Fisher’s exact tests were applied to compare categorical variables between patients with positive and negative tests. The kappa statistic was used to evaluate the agreement between readers^20^ and was interpreted according to Landis and Koch.^21^ When a kappa of 0.4 was reached, accuracy was evaluated. Diagnostic accuracy parameters were calculated by crosstabulation and included the following: sensitivity, specificity, and positive (LR+) and negative (LR−) likelihood ratios. We used the intraclass correlation coefficient (ICC) to evaluate the agreement of the two readers with regard to the RALE score.^22^ The AUC^23^ was used to evaluate the ability of the RALE score to discriminate between COVID-19 and control patients and between poor and favorable outcomes in COVID-19 patients. The discriminatory ability was also evaluated in patients who presented at early (0–2 days), intermediate (3–5 days), and late (≥6 days) time points from symptom onset. For prognostic ability, we used a RALE score cutoff threshold of 5. All statistical tests were two-sided, and p<0.05 was considered statistically significant. For statistical analyses, we used SPSS software (IBM SPSS Statistics for Windows, IBM Corp., Armonk, NY).

## RESULTS

### Patient Characteristics

During the study period, 105 patients had positive RT-PCR results and had a CXR, and 78 patients had repeated negative results and had a CXR. After excluding patients who had the RT-PCR ordered due to an abnormal CXR (n = 1 COVID-19 patient, n = 3 control patients), our study group included 104 COVID-19 patients (men 78/104, 75%, mean age 57.0, SD 15.7 years) and 75 control patients (men 38/75, 51%, mean age 65.6, SD 21.4 years) (Figure 1). Table 1 shows patient characteristics and outcomes with a comparison of COVID-19 to control and non-severe to severe COVID-19 patients.

The overall number of ED visits at our hospital during the study period was 8025 (all causes). The number of new cases of COVID-19 in Israel during the study period (26 days) was 5699. The number for the period immediately preceding was 17. The number for the period immediately ensuing was 9723. These numbers show that our study took place at the beginning of the first wave of COVID-19 in Israel.

The mortality rate in the control group was significantly higher: 27% (20/75) vs 7% (7/104) in COVID-19 patients (P<0.001). Heart disease and active cancer were more common in the control group. Heart disease was present in 44% (n = 33/75) of the control patients compared to 16% (n = 17/104) of COVID-19 patients (p<0.001). Active cancer, defined as malignancy in the prior 12 months, was present in 24% (n =18/75) of the control patients compared to 4% (n = 4/104) of COVID-19 patients (p<0.001). There was no significant difference (p>0.05) in the prevalence of other comorbidities between the control and COVID-19 patients: diabetes mellitus (34%, 29%); hypertension (32%, 38%); obesity (12%, 13%); dyslipidemia (35%, 29%); smoking (19%, 11%); respiratory disease (13%, 7%); and chronic renal failure (7%, 7%).

### Chest Radiograph Technique

Most COVID-19 patients underwent a PA CXR (78/104, 75%) in a dedicated radiography room of the Corona Section emergency department (ED), while most control group patients underwent an AP CXR (45/75, 60%) (p<0.001). Among the COVID-19 patients, most patients with non-severe disease had a PA CXR (64/75, 85%), while most patients with severe disease had an AP CXR (15/29, 52%) (p<0.001). The majority of both the COVID-19 and control groups underwent computerized radiography (CR) (77/104, 74% and 50/75, 67%, respectively) (p=0.284). Similar proportions were observed between patients with non-severe and severe disease.

### Radiographic Findings

The identification of any opacity on CXRs had a moderate interobserver agreement (kappa = 0.408). When assuming that any parenchymal lung opacity could represent COVID-19 pneumonia, the diagnostic accuracy for the diagnosis of COVID-19 for both readers was sensitivity (R1-87%; R2-69%) and specificity (R1-25%; R2-27%), and both LR+ and LR− showed the poor diagnostic performance of CXRs for COVID-19, as most crossed or included 1 (Table 2). See the supplemental table for a summary of pulmonary opacities identified at different timeframes from symptom onset.

The predominance and distribution of opacities, laterality, change from previous radiograph, and expert opinion with regard to COVID-19 status had only a fair agreement between readers (kappa = 0.399, 0.248, 0.372, 0.352, 0.249, respectively); hence, accuracy was not evaluated. The presence of a definite pleural effusion had almost perfect interobserver agreement (kappa = 0.833). The accuracy parameters of the presence of a pleural effusion for the diagnosis of COVID-19 were as follows: sensitivity (R1 and R2 - 0.01%), specificity (R1-81%; R2-77%), and very low positive likelihood ratio (LR+) (R1-0.05; R-0.04); thus, the presence of definite pleural effusion at presentation makes the diagnosis of COVID-19 very unlikely (see Table 2).

With regard to RALE scoring, 103 CXRs were available in the COVID-19 group after excluding one CXR due to pleural effusion, and 55 CXRs were available in the control group after excluding CXRs with the following overshadowing radiopaque abnormalities: pleural effusion (n = 17); lung cancer (n = 1); multiple metastases (n = 1); and calcified pleural plaques (n =1) (Figure 1). The RALE score interobserver reliability was moderate to good, with an ICC of 0.745 (0.665 – 0.806, p<0.001). See Table 3 for the AUC assessment summary.

**Figure 3:**
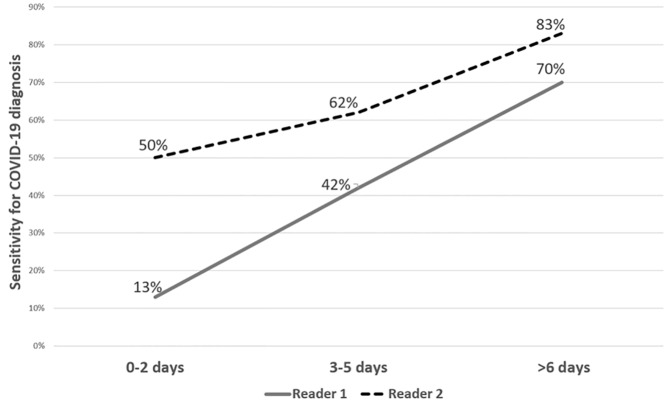
Sensitivity for RALE score threshold of 5 for the diagnosis of coronavirus disease 2019 in patients presenting at different timeframes from symptom onset.

**Figure 4:**
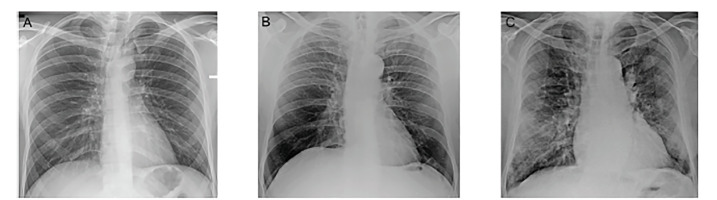
Radiographs of three different COVID-19 patients who presented with fever and cough at different time frames from disease onset. (a) A 32-year-old man who presented one day after symptom onset. Radiograph assessment of lung edema (RALE) scoring: reader 1: 0; reader 2: 0. (b) A 64-year-old man who presented three days after symptom onset. RALE scoring: reader 1: 1; reader 2: 2. (c) A 73-year-old man who presented seven days after symptom onset. RALE scoring: reader 1: 6; reader 2: 6.

The AUC for all patients (overall) showed no significant difference from sheer chance (R1- p = 0.010; R2- 0.865). The evaluation of the discriminatory ability of the RALE score in patients who presented early (0–2 days) showed an inverse correlation with COVID-19 diagnosis. Simply put, in patients presenting within 0–2 days of symptom onset who were clinically suspected of having COVID-19, pulmonary opacities were more likely to be due to a diagnosis other than COVID-19. For patients presenting within three to five days from symptom onset, only R1 achieved statistical significance, while for patients presenting more than six days from symptom onset, both readers reached significant discrimination ability. Thus, for patients presenting later after symptom onset, especially from day six, the higher the RALE score, the more likely a diagnosis of COVID-19. An example is seen in Figure 3, showing the sensitivity of the RALE score with a threshold of 5 for the diagnosis of COVID-19 increasing as the patients arrive later in the disease course. See Figure 4 for CXR examples of patients presenting at different timeframes from symptom onset.

When the RALE score was evaluated as a prognostic indicator within the COVID-19 patient group, both readers had statistically significant discriminatory accuracy for severe disease and poor outcomes (Table 3).

When a RALE score of 5 was used as a threshold for severe disease and for poor outcome, sensitivity was moderate to good, and specificity was moderate. However, LRs were encouraging, as LR+ ranged from 2.21 to 2.59 and LR− ranged from 0.10 to 0.45 (supplemental table). Hence, a RALE score <5 in COVID-19 patients at presentation substantially reduces the odds of having severe COVID-19 or poor outcome (intensive care unit hospitalization, intubation, or death), whereas a RALE score ≥5 substantially increases those odds.

## DISCUSSION

In this study we assessed the diagnostic value of the initial CXR for diagnosing COVID-19 in patients clinically suspected of having COVID-19, as well as the prognostic value of this CXR in COVID-19 patients. The study took place in a single hospital in Israel at the beginning of the COVID-19 pandemic first wave. Our study showed that the reliability of radiographs is only moderate for any opacity and moderate to good for the RALE score. Overall, chest radiography was found not to be a valid diagnostic tool for COVID-19. However, the diagnosis of COVID-19 pneumonia by CXRs reached significant diagnostic accuracy when performed at least six days after symptom onset. For patients presenting early (0–2 days from symptom onset), a normal or near-normal CXR is more likely to be seen in a patient with COVID-19, although opacities early in the disease course do not completely rule out this condition. The presence of a definite pleural effusion indicates that the diagnosis is unlikely to be COVID-19. More extensive lung opacities are associated with poor outcome in COVID-19 patients.

Previous COVID-19 studies mainly concentrated on computed tomography (CT) findings and indicated that opacities are usually bilateral, with a peripheral distribution and lower zones predominance.^24^ We found only fair agreement with regard to the opacity predominance, distribution, and laterality, which probably relates to the lower sensitivity of CXRs compared with CT for pulmonary opacities. A previously published study reported 69% sensitivity for diagnosis on the baseline CXR,^9^ similar to our findings. On the other hand, we found that this high sensitivity had a trade-off with low specificity, which represents the reader’s avoidance of false-negative results, offsetting with more false-positive results. This observation can only be made with a control group. This is in contrast to previous studies that did not have a control group^9^,^11^ and were only able to assess sensitivity. Moreover, LRs showed the CXR is ineffective in the ED setting as it failed to meaningfully change the estimation of disease probability from pretest to posttest. This, at the very least, raises doubts about the utility of the CXR as a triage tool. It is perhaps not surprising that the quantification of pulmonary opacities, as performed in our study with the RALE score, was not useful for assessing the entire cohort when trying to distinguish between patients with and without COVID-19, but when interpreted in the context of time from symptom onset, the accuracy improved.

Highly experienced radiologists’ expert opinions for guessing COVID-19 status were not reliable and did not reach a high enough interobserver agreement to discuss the accuracy parameters. However, poor interobserver agreement regarding specific disease status on CXRs was documented in previous studies.^25^,^26^

Despite the limited role of imaging in the diagnosis of COVID-19 as expressed by leading societies worldwide,^12^,^13^,^27^ the CXR is still the recommended imaging tool for any patient presenting at the ED with an acute respiratory illness.^28^ Future COVID-19 patients will continue to have CXRs at presentation before their disease status is known to the referring clinicians. To complicate matters, even in the ideal setting, when RT-PCR is available and results are delivered within minutes to hours, the sensitivity of the RT-PCR for SARS-CoV2 is poor,^29^ leaving emergency clinicians with a dilemma as to how to manage patients with non-specific presenting symptoms suggestive of COVID-19 with a negative initial RT-PCR test. This dilemma emphasizes the need to maximize available knowledge in the ED setting. Time from symptom onset is available data in this setting, and applying it to CXR interpretation may improve diagnostic accuracy.

Despite not being recommended for diagnosis of COVID-19, the CXR is a tool used for the risk stratification of patients with COVID-19 and is often used as an aid to decision-making with regard to discharge vs hospitalization and the amount of close monitoring needed for specific patients.^13^,^18^,^20^ Our study validates this approach and shows that the amount of pulmonary opacities, as quantified by the RALE score, correlates with poor outcome. The knowledge gained from this study allows for a better understanding of the diagnostic and prognostic value of CXRs in COVID-19 patients and can aid emergency physicians in clinical decision-making. The added information can also serve educators and future researchers in understanding the strengths and weaknesses of CXRs, as this “classic” imaging modality is also the most frequently performed.

## LIMITATIONS

This study has several sources of bias. Differential verification bias (double gold standard bias)^17^ was present in our study, as we selected patients with only one RT-PCR test for the COVID-19 group, whereas we selected only patients with two negative RT-PCR tests for the control group. Lack of clinical follow-up to confirm the absence of COVID-19 precluded incorporation of this patient population with only one negative test into our study. In our opinion, the bias reduced specificity, as the patients in the control group were sicker with almost four times the mortality rate and a higher prevalence of heart disease and active cancer. Thus, the patients in the control group probably had more lung opacities than would be expected in the general population.

Similarly, spectrum bias potentially influenced our results because the control group was enriched with many “sickest of the sick,” whose clinical condition influenced the decision to repeat the test and, hence, could underestimate the specificity.^17^ Even though this methodology is well accepted,^16^ and the motivation was to ensure having only truly non-COVID-19 patients in the control group, the trade-off eliminated many non-COVID-19 patients who might have had less remarkable radiographs. All these biases do not impact the results regarding prognosis, as these did not relate to the control group.

The study’s results can be generalized to the ED setting. In a community setting, in which fewer non-COVID-19 patients have competing conditions, LRs will move further away from 1, and the test will appear more useful.^31^

## CONCLUSION

Chest radiography was found not to be a valid diagnostic tool for COVID-19. However, sensitivity increased in patients presenting later in the disease course. When presenting early, a normal or near-normal CXR is more likely in COVID-19. When a pleural effusion is present, the diagnosis is unlikely to be COVID-19. Furthermore, more extensive lung opacities at presentation are associated with poor outcome in COVID-19 patients. Thus, patients with more than minimal opacities should be monitored closely for clinical deterioration. This clinical application of chest radiography is its greatest strength in COVID-19 as it impacts patient care.

## Supplementary Information



## Figures and Tables

**Table 1:** Patient characteristics and outcomes with comparison of COVID-19 to control and non-severe to severe COVID-19.

Variable	COVID-19 patients	Control	P-value	Non-severe COVID-19	Severe COVID-19	P-value
Gender (Men)	78/104 (75%)	38/75 (51%)	0.001	56/75 (75%)	22/29 (76%)	0.900
Age (years)*	57.0 ±15.7	65.6 (21.4)	0.058	55.64 (15.1)	60.45 (16.9)	0.163
Discharge from ED	31/104 (30%)	13/75 (17%)	0.157	31/75 (41%)	0/29 (0%)	<0.001
Ward hospitalization	59/104 (57%)	51/75 (68%)	0.157	44/75 (59%)	15/29 (52%)	<0.001
ICU	14/104 (13%)	11/75 (15%)	0.157	0/75 (0%)	14/29 (48%)	<0.001
In-hospital mortality	7/104 (7%)	20/75 (27%)	<0.001	0/75 (0%)	7/29 (24%)	<0.001
Intubated	14/104 (13%)	17/75 (23%)	0.108	0/75 (0%)	14/29 (48%)	<0.001

Unless otherwise specified, data are numbers of patients, with frequency in parentheses.

* mean ±SD

**Table 2:** Reliability and accuracy of different radiographic characteristic and experts’ best guess to predict COVID-19 status.

Radiographic variable	Kappa	Sensitivity (95% CI)	Specificity (95% CI)	LR+ (95% CI)	LR− (95% CI)
Any opacity (overall)	0.408				
Reader 1		0.87 (0.78–0.92)	0.25 (0.16–0.37)	1.16 (1.00–1.35)	0.53 (0.30–0.94)
Reader 2		0.69 (0.59–0.78)	0.27 (0.27–0.17)	0.95 (0.94–1.14)	1.15 (0.80–1.65)
Opacity predominance	0.399				
Opacity distribution	0.248				
Laterality*	0.372				
Definite pleural effusion	0.833				
Reader 1		0.01 (<0.01–0.06)	0.81 (0.70–0.89)	0.05 (0.01–0.38)	1.22 (1.19–1.25)
Reader 2		0.01 (<0.01–0.06)	0.77 (0.66–0.86)	0.04 (<0.01–0.31)	1.28 (1.25–1.32)
Change**	0.352				
Experts’ best guess	0.249				

When interobserver reliability did not reach a kappa of 0.4, diagnostic accuracy parameters were not calculated..

* Laterality = bilateral or unilateral.

** Change = change from previous radiograph when comparison was available.

*LR+*, positive likelihood ratio; *LR−*, negative likelihood ratio; *CI*, confidence interval.

**Table 3:** Categorization by RALE score for diagnosis and prognosis of COVID-19 by receiver operator characteristics curve analysis.

Radiographic variable	AUC (95% CI)	P-value
RALE score for diagnosis*		
All patients		
Reader 1	0.625 (0.529 – 0.721)	0.010
Reader 2	0.508 (0.412 – 0.605)	0.865
Days 0–2		
Reader 1	0.290 (0.136 – 0.443)	0.023
Reader 2	0.249 (0.095 – 0.402)	0.007
Days 3–5		
Reader 1	0.741 (0.567 – 0.916)	0.025
Reader 2	0.561 (0.351 – 0.771)	0.570
Days 6≥		
Reader 1	0.738 (0.571 – 0.905)	0.002
Reader 2	0.704 (0.551 – 0.856)	0.009
RALE score for prognosis**		
Severe COVID-19		
Reader 1	0.825 (0.742 – 0.907)	<0.001
Reader 2	0.755 (0.651 – 0.859)	<0.001
Poor outcome		
Reader 1	0.837 (0.736 – 0.937)	<0.001
Reader 2	0.772 (0.636 – 0.907)	0.001

Data are area under the receiver operating characteristic curve (AUC), with 95% confidence interval in parentheses. The RALE score intraclass correlation coefficient (ICC) was 0.745 (95% CI, 0.665–8.086), p-value <0.001.

* Included only patients without radiopaque overshadowing abnormalities (N = 158).

** Included COVID-19 patients without radiopaque overshadowing abnormalities (n = 103).

*CI*, confidence interval; *COVID-19*, coronavirus disease 2019; *RALE*, radiographic assessment of lung edema.
