# Supplementary material for: Diagnostic and Prognostic Value of Chest Radiographs for COVID-19 at Presentation
Source: West J Emerg Med. 2020 Aug 17;21(5):1067–75. doi: 10.5811/westjem.2020.7.48842 (PMC7514404; doi:10.5811/westjem.2020.7.48842)
Supplement: Supplementary file 1 [file wjem-21-1067-s001.docx]

| Radiographic variable | Sensitivity  (95% CI) | Specificity  (95% CI) | LR+  (95% CI) | LR-  (95% CI) |
| --- | --- | --- | --- | --- |
| COVID-19 Diagnosis (overall)^*^ |  |  |  |  |
| Reader 1 | 0.51 (0.41-0.61) | 0.55 (0.43-0.66) | 1.12 (0.82-1.53) | 0.90 (0.72-1.11) |
| Reader 2 | 0.69 (0.59-0.78) | 0.57 (0.74-0.38) | 0.94 (0.78-1.14) | 1.15 (0.80-1.65) |
| COVID-19 Diagnosis (days 0-2)^*^ |  |  |  |  |
| Reader 1 | 0.13 (0.02-0.40) | 0.49 (0.31-0.66) | 0.24 (0.06-0.92) | 1.80 (1.41-2.31) |
| Reader 2 | 0.50 (0.26-0.74) | 0.33 (0.20-0.48) | 0.59 (0.35-0.98) | 3.30 (1.145-7.51) |
| COVID-19 Diagnosis (days 3-5)^*^ |  |  |  |  |
| Reader 1 | 0.42 (0.30-0.61) | 0.75 (0.43-0.93) | 1.81 (0.64-5.10) | 0.73 (0.53-1.01) |
| Reader 2 | 0.62 (0.47-0.76) | 0.42 (0.16-0.71) | 1.06 (0.62-1.81) | 0.91 (0.49-1.69) |
| COVID-19 Diagnosis (>6 days)^*^ |  |  |  |  |
| Reader 1 | 0.70 (0.54-0.82) | 0.53 (0.35-0.71) | 1.49 (0.97-2.29) | 0.57 (0.35-0.94) |
| Reader 2 | 0.83 (0.68-0.92) | 0.33 (0.18-0.53) | 1.24 (0.93-1.65) | 0.52 (0.24-1.12) |
| Severe COVID-19^**^ |  |  |  |  |
| Reader 1 | 0.90 (0.72-0.97) | 0.64 (0.52-0.75) | 2.49 (1.80-3.45) | 0.16 (0.05-0.48) |
| Reader 2 | 0.62 (0.42-0.79) | 0.76 (0.65-0.85) | 2.59 (1.58-4.23) | 0.45 (0.31-0.80) |
| Poor outcome^**^ |  |  |  |  |
| Reader 1 | 0.94 (0.69-1.00) | 0.57 (0.46-0.68) | 2.21 (1.69-2.90) | 0.10 (0.02-0.70) |
| Reader 2 | 0.71 (0.44-0.89) | 0.72 (0.62-0.81) | 2.56 (1.62-4.05) | 0.41 (0.19-0.86) |
| Poor outcome was defined as ICU hospitalization, intubation or death.  Severe COVID-19 was classified based on respiratory distress (≥30 breaths/min) or oxygen saturation ≤93% on room air^18^  *Included only patients without radiopaque overshadowing abnormalities.  ** Included COVID-19 patients without radiopaque overshadowing abnormalities.  *CI,* confidence interval; *ICU*, intensive care unit. | | | | |

**Supplemental table.** Diagnostic and prognostic value of the RALE score threshold of 5.
